# Evaluation of a Rural Emergency Medical Service Project in Germany: Protocol for a Multimethod and Multiperspective Longitudinal Analysis

**DOI:** 10.2196/14358

**Published:** 2020-02-14

**Authors:** Camilla Metelmann, Bibiana Metelmann, Dorothea Kohnen, Clara Prasser, Rebekka Süss, Julia Kuntosch, Dirk Scheer, Timm Laslo, Lutz Fischer, Joachim Hasebrook, Steffen Flessa, Klaus Hahnenkamp, Peter Brinkrolf

**Affiliations:** 1 Clinic for Anaesthesiology University Medicine Greifswald Greifswald Germany; 2 zeb.business school Steinbeis University Berlin Münster Germany; 3 Chair of General Business Administration and Health Management University of Greifswald Greifswald Germany; 4 District of Vorpommern-Greifswald Greifswald Germany; 5 Communal Rescue Services District of Vorpommern-Greifswald Greifswald Germany

**Keywords:** resuscitation, telemedicine, mHealth, smartphone-based alerting, emergency medical services, mobile applications

## Abstract

**Background:**

German emergency medical services are a 2-tiered system with paramedic-staffed ambulances as the primary response, supported by prehospital emergency doctors for life-threatening conditions. As in all European health care systems, German medical practitioners are in short supply, whereas the demand for timely emergency medical care is constantly growing. In rural areas, this has led to critical delays in the provision of emergency medical care. In particular, in cases of cardiac arrest, time is of the essence because, with each passing minute, the chance of survival with good neurological outcome decreases.

**Objective:**

The project has 4 main objectives: (1) reduce the therapy-free interval through widespread reinforcement of resuscitation skills and motivating the public to provide help (ie, bystander cardiopulmonary resuscitation), (2) provide faster professional first aid in addition to rescue services through alerting trained first aiders by mobile phone, (3) make more emergency physicians available more quickly through introducing the tele-emergency physician system, and (4) enhance emergency care through improving the cooperation between statutory health insurance on-call medical services (German: *Kassenärztlicher Bereitschaftsdienst*) and emergency medical services.

**Methods:**

We will evaluate project implementation in a tripartite prospective and intervention study. First, in medical evaluation, we will assess the influences of various project measures on quality of care using multiple methods. Second, the economic evaluation will mainly focus on the valuation of inputs and outcomes of the different measures while considering various relevant indicators. Third, as part of the work and organizational analysis, we will assess important work- and occupational-related parameters, as well as network and regional indexes.

**Results:**

We started the project in 2017 and will complete enrollment in 2020. We finished the preanalysis phase in September 2018.

**Conclusions:**

Overall, implementation of the project will entail realigning emergency medicine in rural areas and enhancing the quality of medical emergency care in the long term. We expect the project to lead to a measurable increase in medical laypersons’ individual motivation to provide resuscitation, to strengthen resuscitation skills, and to result in medical laypersons providing first aid much more frequently. Furthermore, we intend the project to decrease the therapy-free interval in cases of cardiac arrest by dispatching first aiders via mobile phones. Previous projects in urban regions have shown that the tele-emergency physician system can provide a higher availability and quality of emergency call-outs in regular health care. We expect a closer interrelation of emergency practices of statutory health insurance physicians with the rescue service to lead to better coordination of rescue and on-call services.

**International Registered Report Identifier (IRRID):**

DERR1-10.2196/14358

## Introduction

### Status of German Emergency Care

German emergency medical services (EMSs) are a 2-tiered system using ambulances and emergency response vehicles. Ambulances are staffed by 2 paramedics, whereas emergency response vehicles are staffed by a paramedic and an emergency physician [1]. For life-threatening emergencies, both ambulances and emergency response vehicles are alerted, whereas less-critical situations require only an ambulance [2], which relates to approximately half of all cases [3,4].

German EMSs are facing the challenge of responding to constantly rising numbers of call-outs with fewer and fewer emergency physicians—this is especially difficult in rural areas [5-8]. Simultaneously, legal requirements of an optimal response time from the rescue service are becoming difficult to meet. In fact, the rescue service law for the state of Mecklenburg-Vorpommern (Mecklenburg-West Pomerania) requires statewide compliance with the response time of 10 minutes for the first response vehicle on the scene [9,10] and 15 minutes for emergency physician. The response time (German: *Hilfsfrist*) is defined as the time between alerting the rescue services and their arrival on a passable road at the place of need. According to the legal requirements, the response time must be guaranteed in 95% of cases in cities of more than 20,000 inhabitants or in 90% of cases in rural areas.

For the district of Vorpommern-Greifswald, a region that belongs to the state of Mecklenburg-Vorpommern, calculations by the Chair of General Business Administration and Health Care Management at the University of Greifswald revealed that in 14 of the 36 areas, the emergency physician did not arrive within 5 minutes of the ambulance in 90% of cases (based on the official figures for 2014) [11]. Each year, numerous tourists visit the popular holiday region on a seasonal basis, leading to increased cases of emergencies and that also need to be guaranteed support in an emergency. This additional burden was not included in the calculations of simulations.

Call-outs due to cardiac emergencies are particularly urgent as, for example in cardiac arrest, vital organs such as the brain are no longer supplied with oxygen; the longer the time to first resuscitation measures, the lower the rate of survival [12]. Accordingly, compared with urban areas, in rural regions the chance of surviving a cardiac arrest substantially decreases due to longer travel time to the emergency site [13]. During a medical emergency, it is not only the response time of professional emergency services that is critical, but also the therapy-free interval—that is, the time from occurrence of the emergency to the start of qualified aid assistance [14]. To realign emergency medicine in rural areas, the project Land|Rettung (English: Rural|Rescue) is focusing on 2 solution approaches: (1) structuring professional rescue services using information and communication technologies to reduce the therapy-free interval, and (2) introducing layperson resuscitation, hence bridging the longer arrival times of professional services during an emergency situation [14,15].

### Project Land|Rettung

**Figure 1:**
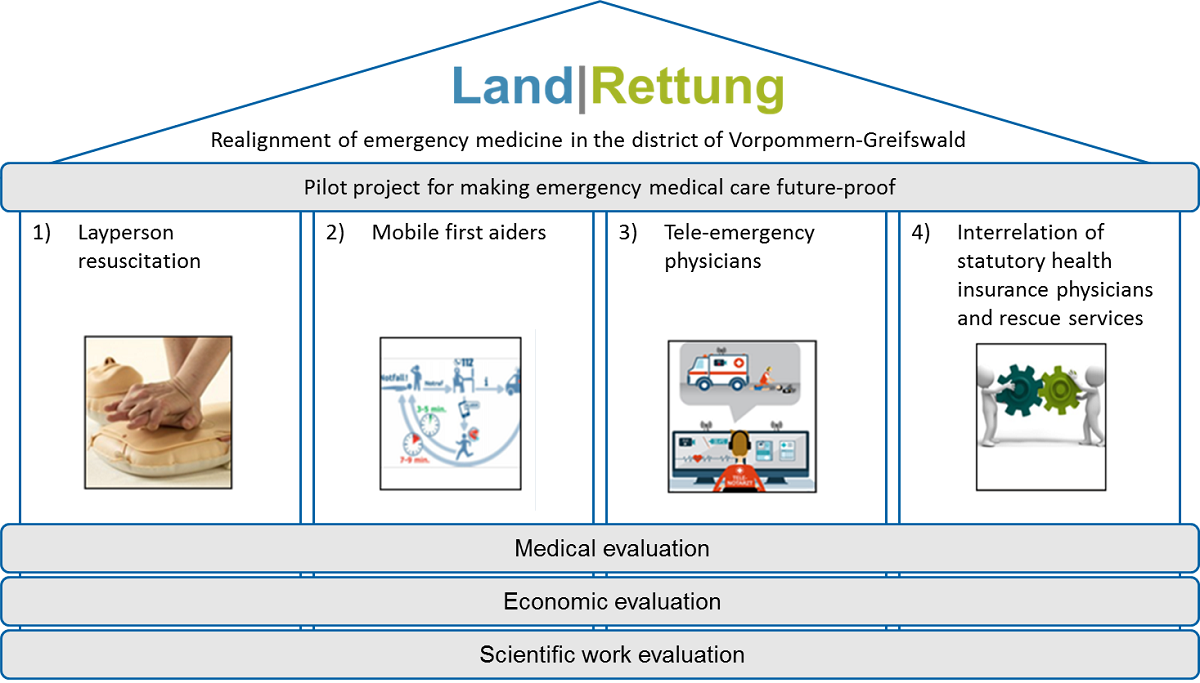
The project Land|Rettung (English: Rural|Rescue) and its 4 pillars.

To realign emergency services, Land|Rettung follows a multimethodological approach based on 4 pillars that also are the project’s objectives (see Figure 1): (1) reduce the therapy-free interval through a widespread reinforcement of society’s resuscitation skills and motivation to provide assistance using targeted training methods (ie, bystander cardiopulmonary resuscitation [CPR]), (2) provide fast professional first aid in addition to rescue services through the involvement of specially trained first aiders alerted via a mobile phone app (Land|Retter app), (3) provide faster and greater availability of emergency physicians through the establishment of the tele-emergency physician (TEP) system, and (4) more closely interlock emergency care through the realignment of emergency practices of statutory health insurance physicians and EMSs.

Being the first of its kind in Germany, this 4-module project will lead to a joint concept developed and established by all groups involved in emergency care: state government, districts, health insurers, rescue services, hospitals, and statutory health insurance physicians. Involving all relevant parties in the process of realigning emergency medicine in rural areas will also allow for the concept’s transformation to other regions in Mecklenburg-Vorpommern and other states.

### Reducing the Therapy-Free Interval

As part of the project, we are implementing 2 interventions to reduce the therapy-free interval in cases of cardiac arrest.

First, in a cardiac emergency, medical laypersons who are in the immediate vicinity of the person affected are an important factor. Between 67% and 84% of cardiac arrests occur at home, and approximately that many are witnessed by bystanders [16,17]. Bystander CPR—that is, CPR given before the arrival of medical emergency personnel—is performed in Germany in about 37% (in 2017) of cases, which is low compared with other countries [18]. Therefore, not only knowledge but also the willingness to perform resuscitation among the population has to be increased [19]. Chest compressions ensure that oxygen is transported to the brain. Laypersons starting resuscitation can bridge the time until EMSs arrive and thus increase the likelihood of survival [20]. Resuscitation measures by the EMS lead to twice as good a result when layperson resuscitation has been conducted beforehand [21,22]. Resuscitation training in communities is associated with higher survival rates [23]. Layperson resuscitation is already promoted through Germanywide projects such as “Ein Leben retten” (save a life) [24]. Accordingly, one key objective of this project is the targeted promotion of layperson resuscitation.

Second, studies have proven that chest compressions performed by a highly trained first aider have a greater chance of success than those performed by a medical layperson [25,26]. Based on their professional or voluntary qualifications, these first aiders can include physicians, dentists, paramedic and rescue personnel, nursing personnel, medical service staff, company paramedics, fire department personnel, medical assistants, and students of medicine and dentistry. Their involvement can improve treatment of patients with cardiac arrest even before the EMS arrives [27,28]. The fact that approximately 11% of the German population is medically trained [29] increases the probability that a person trained in resuscitation is in the immediate proximity of a person with cardiac arrest. To involve and engage as many medical professionals as possible, we have developed a mobile phone–based app that connects the private mobile phone of the trained first aider with the alert system of the rescue directing center. As part of the intervention, first aiders are alerted by mobile phone and dispatched to resuscitate patients experiencing cardiac arrest nearby. Due to their closer proximity, first aiders could arrive on-site before the ambulance and start resuscitation [30].

### Earlier Treatment by an Emergency Physician

In addition to first aid interventions, the project focuses on structuring professional rescue services using a variety of information and communication technology tools. To assure and to improve the quality of emergency care in districts covering large areas with a low population density, suitable strategies need to be developed guaranteeing continuous availability of both technical and individual resources.

**Figure 2:**
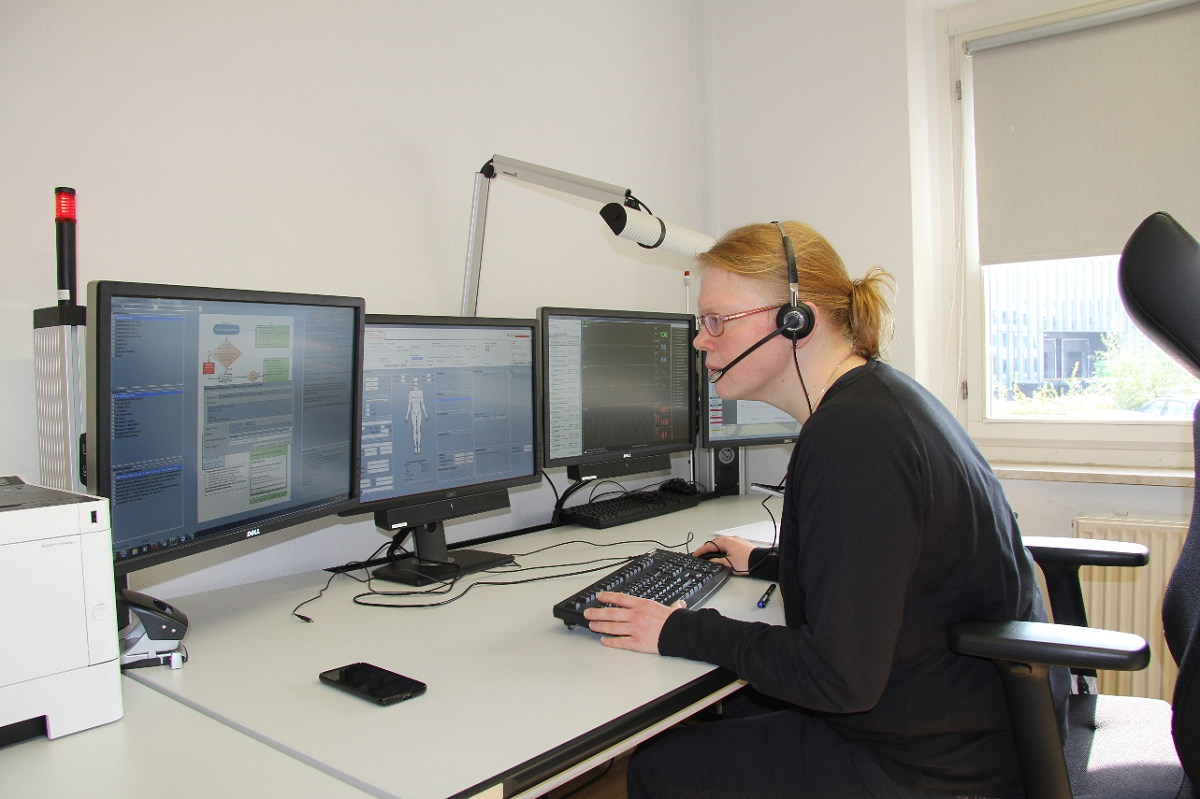
Tele-emergency physician station.

Telemedicine in emergency medical care, such as a TEP system, can contribute to ensuring high-quality medical care in rural areas [31,32]. In emergency services with moving vehicles, however, the standard requirements for telemedicine are much higher than in areas where data transfer solely runs over local networks (eg, over a local area network or Wi-Fi) between physicians in the same field [33-35]. Even the technical aspect of data connection via mobile transfer media is challenging in rural areas. Equally challenging are the issues of new structures in work organization, employment law, liability law, and funding [36]. Previous pilot projects have been mainly oriented toward technical aspects but rarely have been translated into practice. There are, however, some exceptional projects where manufacturers of medical device technology and telecommunication specialists have developed devices in cooperation with rescue service personnel, emergency physicians, and emergency departments. These devices have also been trialed in test operations to evaluate their effectiveness and safety in regular operations [37-40]. One example is the Aachen TEP system. This well-engineered concept, introduced in 2014, is composed of highly specialized training, selected medical device components, optimized transmission technology, and customized software modules [31,34,41]. Our Land|Rettung project is technically grounded in the Aachen TEP system, and we work closely together on technical realization and improvements (see Figure 2).

### Interlinking of Statutory Health Insurance Physicians and Rescue Services

In Germany, prehospital emergency medical care is multilayered. Depending on the urgency of the medical problem, patients can contact 2 different systems: (1) the EMS and (2) statutory health insurance physicians [42-44]. While EMSs provide the care of patients with life-threatening or severe illnesses, statutory health insurance physicians take over the tasks of general practitioners outside regular hours by means of on-call medical services. Up to now, these 2 systems have operated independently of each other, as both are accessible through specifically installed telephone hotlines. While the EMS is easily accessible and is handling an increasing workload with a growing number of inadequate uses, statutory health insurance physicians are experiencing staffing problems and areas that are too large to cover [45]. In addition, emergency rooms are facing a growing burden of patients who are not hospitalized by a doctor, but who are seeking advice on their own initiative [46,47]. Overall, these 2 separate systems need to cooperate and should be interlinked [48] in order to achieve adequate patient control and optimal deployment of human resources.

### Objective

Here we provide an overview of a rural EMS project in Germany, which is based on a multimethod and multiperspective longitudinal analysis.

## Methods

### A Multimethodological Approach From Different Perspectives

We will assess and evaluate the implementation and results of the project through a tripartite prospective and intervention study from (1) a medical, (2) an economic, and (3) a work and organizational perspective. First, as part of the medical evaluation, we will assess the influences of various project measures on the quality of care using multiple methods. For this, we will review the emergency services logs for general quality of monitoring and documentation, quality of analgesia and pain reduction, quality of diagnoses, and the rate of adverse events. We will gather indicators for tracer diagnoses on the quality of medical treatment and results. We are also planning an analysis of cases of stroke and acute coronary syndrome, regarding how often the tentative diagnosis in prehospital treatment matches with the diagnosis verified in hospital. Second, in the economic evaluation we will include the number of call-outs, distribution between conventional and TEP call-outs, rate of calls for emergency physician reinforcements, actual response times, and overall call-out duration and engagement times for emergency physicians. In addition to the costs and time analyses, we will conduct an outcome analysis to evaluate the TEP system. Third, as part of the work and organizational analysis, we will analyze the necessary changes to work processes, workload, and satisfaction of all affected occupational groups; gather organizational, leadership, and cooperation structures; and assess regional effects, especially regarding cooperation between the occupational groups and the development of joint standards.

To assess and evaluate the project’s results, each evaluation part consists of different procedures. All information relevant for data collection is available from the regularly kept logs for call-out documentation in rescue services (ie, emergency physician logs, TEP logs, and data from computer-aided dispatch), as well as data from hospitals providing follow-up care.

#### Medical Evaluation

The medical analysis will consist of 3 parts (see Table 1). The first part will focus on the documentation and treatment quality of the individual emergency cases. Second, we will assess how often the tentative preclinical diagnoses is concordant with that made by the hospital. Diagnostic capabilities are limited outside a hospital; for example, x-rays and laboratory tests cannot be conducted. For this reason, initial diagnoses made by the EMS remain tentative. As part of the project, we will examine how often the tentative diagnosis can be confirmed and how many false-negative or false-positive decisions are made. Third, we will gather and analyze important figures that characterize the general quality of treatment in rescue services.

**Table 1:** Medical analysis.

Subject of analysis	Criteria
Documentation and treatment quality of each emergency case	General monitoring quality Blood sugar measurements in cases of loss of consciousness Quality of analgesia or pain reduction Documentation quality of general medical history Specific documentation quality of allergies Specific documentation quality of previous medication Quality of diagnosis
Correctness of tentative diagnoses made by the emergency medical service	Patient was treated only by paramedics Patient was treated by paramedics and an emergency physician on-site Patient was treated by paramedics and a tele-emergency physician
General quality of treatment of the emergency medical service	Therapy-free interval for 6 selected tracer diagnoses (stroke, hypertensive emergency, acute coronary syndrome, asthma, exacerbated chronic obstructive pulmonary disease, and polytrauma) Bystander cardiopulmonary resuscitation rate Number of surviving patients who were discharged from hospital after resuscitation

#### Economic Evaluation

Similar to the medical analysis, the economic evaluation generally refers to data obtained from regularly kept logs for case documentation in rescue services, as well as from legally prescribed financial accounting or management accounting for rescue services. Accordingly, the economic evaluation will also consist of 3 areas of analysis (see Table 2). The first part will aim to calculate the total costs for each measure, divided into fixed and variable costs. We will collect data mainly from the EMS documents and contracts with external partners. Based on a break-even analysis, we will compare the total costs of the TEP concept versus standard care to find a cost-efficient solution for future implementation. This will further depend on the number of areas in which emergency care cannot be provided within the prescribed response time. The second part will analyze the influence of various project measures of central parameters of emergency rescue by means of pre-post comparison. After determination, we will compare the respective parameters separately with and without the TEP system being involved. As before, we will obtain data from regularly gathered call-out data for the rescue service. We will summarize and directly transfer the data before implementation of the project measures at regular intervals. The same applies to the data after implementation of the TEP system. We will check the EMS data for plausibility, calculate the necessary parameters, and compare the results specifically per group: pre-post, times of year, times of the day, areas, etc. The third part will focus on technical and temporal parameters of the first aider app. We will gather data from EMS logs and from app logs. We will focus on technical disruption of the TEP system and gather data from the TEP system logs.

**Table 2:** Economic analysis.

Subject of analysis	Criteria
Determination of costs (fixed and variable) for each measure Comparison of total costs of the tele-emergency physician concept and standard care	Investment costs Ongoing annual operating costs
Influence of various measures on the central parameter of emergency rescue	Response time Personnel engagement time during call-outs Transfer times Operational distances Total operating times Rate of subsequent calls for reinforcement of emergency physicians
Technical and temporal parameters of the first aider app and the tele-emergency physician system	Time between arrival of the mobile first aider and the rescue service Numbers of technical breakdowns of the tele-emergency physician system

In addition to the costs and time analyses, we will analyze patient outcomes based on the data resulting from the application of the project measures. Our main focus will be on the TEP system introduced at the start of the project. Based on the individual care pathways after a telemedically supported emergency treatment, we will compare the costs and the patients’ individually perceived quality of life after the emergency incident versus standard care data. The main objective will be to determine whether telemedical emergency care has an effect on the individual and overall economic costs and whether it may lead to a life-related value for telemedically treated patients. We will gather the data for this evaluation from anonymized databases provided by the Eigenbetrieb Rettungsdienst Vorpommern-Greifswald.

#### Work and Organizational Analysis

The work and organizational evaluation will generally be based on a pre-post comparison and will differentiate 3 parts of the analyses (Table 3). In the first part, we will conduct expert panels at the beginning and at the end of the project to assess 4 clusters in 3 dimensions on a scale from 1 to 10 and importance on a scale from 1 to 10. We will hold the expert rounds for each of the 4 project pillars: (1) improvement in layperson rescue, (2) use of mobile first aiders, (3) the TEP system, and (4) cooperation between EMSs and statutory health insurance emergency care. In the second part, we will conduct a network analysis. Participants from the expert panel and other people nominated by them will anonymously enter their personal contacts and contacts to other organizations into a network matrix. Thus, we will be able to calculate network size, density, and centrality. The third part of the analysis will comprise a competence and transfer analysis based on surveys developed as part of knowledge transfer studies [49].

### Design

This project is a multimethod and multiperspective longitudinal analysis and control group study. We will use a longitudinal approach not only to understand changes from implementing the 4-pillar concept but also, if needed, to intervene at an early stage. The control group design is essential, as insights into the transferability of the concept can only be gathered in this way. We will collect data across all 4 pillars: layperson resuscitation, mobile phone–based first aider alerts, the TEP system, and cooperation of emergency medicine providers. We will conduct pre-post comparisons of data before and after the introduction of the TEP system.

**Table 3:** Work and organizational analysis.

Subject of analysis	Criteria
Evaluating clusters made of (1) service providers, (2) suppliers, (3) training measures, and (4) new technologies in 3 dimensions of competitiveness (ie, regional, intraregional, international) and importance	Basic factors: Infrastructure (building, accessibility, network expansion) Resources (personnel, material, technology) Stakeholders: Suppliers and supporters, cooperation and competition between organizations involved Area of care provision: Care need (eg, deviations in the type and frequency of emergencies) Transfer to other areas of care provision (intraregional cooperation and support)
Regional networks	Centrality (amount of connected links) Density (relationship of maximum possible links to actually available links)
Competence and transfer analysis Transfer of knowledge and competencies for social networks (ie, explicit, implicit, individual or joint use of knowledge) Type of knowledge and competence transfer (ie, types, sources, instruments)	Accuracy Availability Transparency Accessibility Comprehensibility Up-to-datedness Needs orientation Competence orientation Credibility Use Motivation to share knowledge Subjective perception of the strength of change

### Participants

In the district of Vorpommern-Greifswald, the EMS is alerted to over 40,000 medical emergencies annually (as of 2017), with more than 15,000 alerts going to emergency physicians. Ambulances equipped with technology for the TEP system cover approximately 6500 of those cases annually. To achieve a sufficiently high power for subgroup analyses, we will consider emergency cases occurring over a period of 24 months (October 2017 to October 2019) prospectively within the evaluation process. With regard to general quality parameters for medical care and documentation, we will consider all cases handled by the TEP within the evaluation process as our intervention group. In addition to this overall analysis of TEP cases, we will create subgroups in accordance with the research question of interest. Before project enrollment, we conducted power analyses individually for each subgroup in order to estimate the sample size (ie, the number of patients needed).

As part of the control group, we will analyze emergency cases without telemedical support in a retrospective as well as a prospective design. We will also compare these cases with emergencies handled by the TEP in terms of parameters such as age, sex, and an injury severity score (National Advisory Committee for Aeronautics [NACA] score).

**Figure 3:**
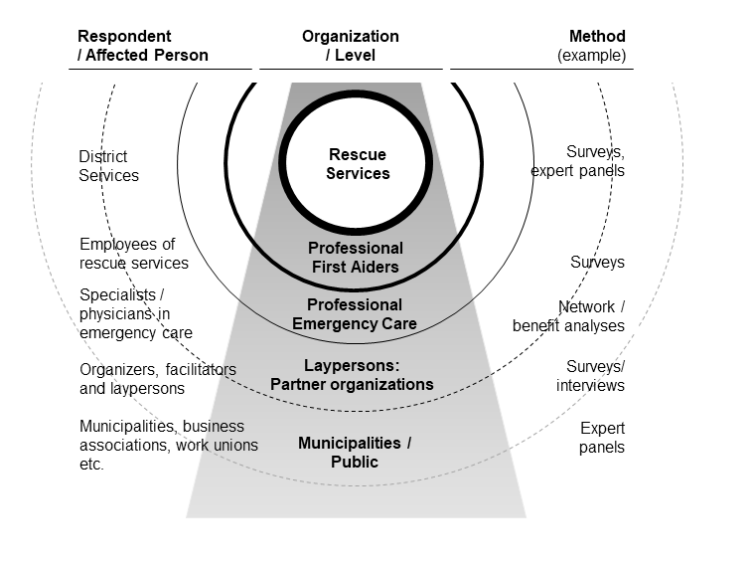
Organizational levels, affected persons, and evaluation methods.

Depending on the organizational level, the work and organizational analysis will focus on different groups being affected by the project measures (see Figure 3). In the first instance, we will consider all employees of the emergency services except for professional first aiders in the analyses. As part of the network and cluster analysis, we will also consider representatives of partner organizations and municipalities.

### Statistical Analysis

We will apply statistical analysis in the medical evaluation to determine differences between the intervention and control groups and to measure the impact of potential influential factors. We will apply multiple regression models to describe and to assess interrelations of cluster strength (eg, emergency systems of 2 regions), network density, and centrality of people and organizations involved in the emergency system, but also to estimate the levels of intensity and time needed to transfer knowledge and competencies between individuals and teams. Overall, this method will help to identify relevant work and organizational factors contributing to the project’s medical and economic success. As regression analyses confirm the expected relationships, we will conduct mutual discriminant analyses to examine the direction of the relationships. More specifically, mutual discriminant analyses will verify whether the coefficient of determination (*r*^2^) is higher when medical or economic parameters are used to predict cluster and network data or levels of transfers, or whether, conversely, work and organizational factors predict economic or medical figures [50,51]. Furthermore, this analysis can be used to assess whether work and organizational factors account for medical and economic performance or whether high medical and economic performance determines the work and organizational factors.

## Results

The project was started in 2017 and enrollment will be completed in 2020. We completed the preanalysis phase in September 2018. Data analysis is underway, and we expect to submit the first results for publication in 2020.

## Discussion

### Relevance

This multimethod and multiperspective longitudinal analysis uses an innovative approach that aims to realign emergency medicine in rural areas and to enhance the quality of medical emergency care in the long term. In particular, its consideration and involvement of all parties engaged in emergency care underlines the project’s uniqueness. We expect the implementation of Land|Rettung to lead to a measurable increase in medical laypersons’ individual skills and motivation to provide resuscitation, with an increasing rate of bystander resuscitation. Experiences from the layperson resuscitation campaign “Ein Leben retten” (save a life) [24], as well as initial training courses in Greifswald, have already indicated that the number of emergencies in which laypersons provided first aid significantly increased after they participated in the relevant courses [52]. Furthermore, the project is intended to decrease the therapy-free interval through the arrival of first aiders at an emergency before the rescue services*.* In some urban areas of Germany, mobile phone apps that alert specially trained first aiders are already being used. Results show that, in up to 57% of all alerts, nearby professional first aiders are able to provide assistance [53]. With regard to our third objective, the TEP system has already shown a higher availability and quality of care as an integral part of the EMS in the city of Aachen, Germany [38,41]. Whereas Aachen offers an excellent infrastructure, with our project we intend to evaluate this concept in a rural area. We expect a closer interrelation between statutory health insurance physicians and the EMS to lead to better coordination of rescue and on-call services.

While the medical evaluation of treatment quality (ie, medical outcome) and the economic evaluation (ie, profitability calculation) will reveal whether transferring the concept is medically and economically feasible, the work and organizational evaluation will assess the transferability of the results. Thus, these 3 aspects of evaluation directly complement one another: while the medical and economic evaluation will check whether the measures implemented make medical and economic sense, the work and organizational evaluation will ensure their sustainability and universal adaptability. The establishment of an evaluation standard would be beneficial in 2 ways: (1) preclinical emergency medicine in the district of Vorpommern-Greifswald will be regularly evaluated with a reasonable effort, and (2) a standard would facilitate the introduction of this new treatment concept in other rural districts and ensure its effectiveness and sustainability.

### Limitations

The project faces some challenges, such as ethical restrictions to conducting such research using digital resources without compromising the ethical or legal credibility and protections for human participants. Another constraint lies in the technical aspect of data connection via mobile transfer media and its limitations, especially in rural areas. With ongoing government investment programs and the interest of the industry, coverage by the Global System for Mobile Communications standard will increase over the next years. Especially in the initial stage of the project and in sparsely populated areas in particular, we recognized that some mobile phone app alerts were not being transmitted, even though specially trained first aiders were within the appl’s radius. The provider’s system data will provide further information on the app’s functionality in different areas of Mecklenburg-Vorpommern. Overall, not all ambulance vehicles could be equipped with the TEP system. Although these systems are proportionally distributed, this factor may restrict the final number of our intervention group.

### Conclusions

We are confident that this project presents an economically sensible and methodically innovative approach that will review the effects of project measures in terms of development of regional treatment networks, and of knowledge and experience transfer between occupational groups and decision makers. All in all, improved cooperation, the formation of a network, and better emergency care should ensure greater treatment provision reliability and increase the region’s appeal. Due to the structural weakness of the region and the high importance of tourism with seasonal peaks, the implementation and evaluation of the concept are important not only for the local population, but also for the many tourists.

## Abbreviations

CPR: cardiopulmonary resuscitation
EMS: emergency medical service
NACA: National Advisory Committee for Aeronautics
TEP: tele-emergency physician
